# Statement of Retraction: Novel Sunifiram carbamate hybrids as potential dual acetylcholinesterase inhibitor and NMDAR co-agonist simulation-guided analogue design and pharmacological screening

**DOI:** 10.1080/14756366.2026.2712703

**Published:** 2026-08-07

We, the Editors and Publisher of the journal *Journal of Enzyme Inhibition and Medicinal Chemistry* have retracted the following article: 

Agha, K. A., Abo-Dya, N. E., Issahaku, A. R., Agoni, C., Soliman, M. E. S., Abdel-Aal, E. H., … Ibrahim, T. S. (2022). Novel Sunifiram-carbamate hybrids as potential dual acetylcholinesterase inhibitor and NMDAR co-agonist: simulation-guided analogue design and pharmacological screening. *Journal of Enzyme Inhibition and Medicinal Chemistry*, *37*(1), 1241–1256. https://doi.org/10.1080/14756366.2022.2068147

Following publication, it was noted that several references in the article contained incorrect information or were unable to be verified. When requested to provide more details to verify the references, the authors confirmed there were fundamental errors present.

The validity of the references is integral to the scientific integrity of an article. As the editor and ­publisher have been unable to determine the validity of the references, we are therefore retracting the article. The authors disagree with the retraction.

We have been informed in our decision-making by our editorial policies and the COPE principles.

The retracted article will remain online to maintain the scholarly record, but it will be digitally ­watermarked on each page as ‘Retracted’. 

